# Non-linear dynamics in ECG: a novel approach for robust classification of cardiovascular disorders

**DOI:** 10.1038/s44325-024-00038-2

**Published:** 2025-02-03

**Authors:** Suraj Kumar Behera, Debanjali Bhattacharya, Ninad Aithal, Neelam Sinha

**Affiliations:** 1 National Institute of Science Education and Research, NISER, Jatni, Bhubaneswar, 752050 Odisha India; 2 Center for Brain Research, Indian Institute of Science (IISc) Campus, CV Raman Avenue, Bangalore, 560012 Karnataka India

**Keywords:** Cardiology, Diseases, Health care

## Abstract

Detecting cardiac disorders from multi-channel ECG has significant implications for cardiac care. Current methods face challenges due to ECG waveform variations by electrode placement, high signal non-linearity, and low millivolt amplitudes. The present study introduces a non-linear analysis approach leveraging Recurrence plot visualizations as the patterned occurrence of well-defined structures, such as the QRS complex, can be exploited effectively using Recurrence plots. Using the Physikalisch-Technische Bundesanstalt dataset from PhysioNet, we examined four cardiac disorder classes- Myocardial infarction, Bundle branch blocks, Cardiomyopathy, Dysrhythmia, and healthy controls, achieving an impressive classification accuracy of 100%. Wilcoxon rank-sum test is performed at 95% C.I. on Recurrence Quantitative Analysis (RQA) features, identifying five features with statistically significant differences across pairs of study groups. Additionally, t-SNE visualizations of latent space embeddings derived from Recurrence plots and RQA features reveal clear separation among cardiac disorders and healthy subjects, underscoring the efficacy of the proposed approach.

## Introduction

Cardiovascular disorders (CVDs) are the leading cause of mortality worldwide, posing significant burdens on public health and healthcare costs^1,2^. CVDs encompass various conditions, each presenting distinct electrocardiographic markers. For example, a Bundle branch block, a disruption in electrical conduction within the heart, causes widened QRS complexes and altered ECG vectors, potentially indicating higher mortality risk in myocardial infarction, heart failure, and other heart block conditions^3^. Cardiomyopathy, also known as heart failure, impairs the heart’s ability to pump blood effectively, leading to complications such as irregular heartbeats. Dysrhythmia, or arrhythmia, involves irregular heart rhythms that increase the risk of stroke, heart failure, and cardiac arrest. Myocardial infarction, commonly referred to as a heart attack, results from a critical loss of blood flow to the heart muscle, making it a prevalent cause of death. Early detection of these disorders through ECG signals is essential to prevent disease progression and enhance patient outcomes. However, manual ECG analysis is time-consuming, leading researchers to explore automated approaches to detect and diagnose CVDs more efficiently. While convolutional neural networks and few-shot learning have been widely used for ECG classification, converting 1D signals into 2D images has also shown promise in improving classification accuracy and understanding data dynamics^4–9^.

*Research Gap:* While existing ECG classification methods like convolutional neural networks and 1D-to-2D transformations offer valuable insights, they often lack the ability to capture the complex, non-linear dynamics inherent in ECG signals, which are critical for understanding CVDs. Non-linear analysis techniques, such as Recurrence plots, offer a unique approach to visualize and quantify the recurrence of states within ECG data, revealing hidden patterns and structural changes relevant for diagnosing cardiac conditions^10–15^. While previous studies have used recurrence quantitative analysis (RQA) to analyze complex behaviors in cardiovascular signals, the integration of these recurrence-based methods with deep learning remains underexplored for CVD classification. Furthermore, existing studies have yet to leverage the latent space representations of autoencoders in combination with Recurrence plots to capture the intricate patterns of CVDs in ECG signals, leaving a gap in efficient, high-accuracy classification techniques. The present study aims to address these gaps by utilizing autoencoder latent-space embeddings of Recurrence plots and RQA for classifying different types of CVDs. By combining the strengths of autoencoders and Recurrence plots, this approach seeks to enhance the detection of non-linear ECG dynamics in different CVDs.

The novelty of this study lies in the utilization of autoencoder latent-space embedding of Recurrence plots and RQA to classify different CVDs. This approach combines the advantages of autoencoders with Recurrence plots, leveraging latent-space representations to enhance the analysis of non-linear dynamics in ECG signals. By embedding Recurrence plots into the latent space of an autoencoder, the study aims to improve the extraction and quantification of complex patterns and features, thereby providing a more robust and accurate classification of various cardiac conditions. This innovative method promises to advance ECG analysis, offering a sophisticated and automated approach for the early detection of CVDs and supporting improved diagnostic and treatment outcomes through non-linear data analysis techniques.

**Fig. 1:**
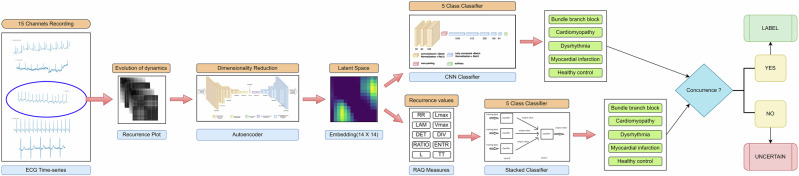
Block diagram of the proposed methodology.

The subsequent sections of the paper are organized as follows: The analysis of the results are discussed in detail in Section “Results” and Section “Discussion”. The dataset description and proposed methodology is explained in Section “Dataset description” and Section “Spatial encoding od ECG using Recurrence plot”, respectively. The block schematic of the proposed methodology is shown in Fig. 1.

## Results

Recurrence plots, due to their symmetric nature, capture and reveal various patterns that reflect the underlying dynamics of the system being studied. These patterns provide insights into how the system evolves over time, making Recurrence plots a valuable tool for analyzing complex time-series data, such as ECG signals. In the present study, we utilize this powerful non-linear analytical technique by combining deep learning, ensemble methods, and rigorous statistical analysis to achieve accurate and robust classification of various CVDs. The proposed approach starts with converting ECG signals into 2D Recurrence plots, which are symmetric representations capturing the temporal dependencies and underlying dynamics of the cardiac system. To handle the high-dimensional data generated from Recurrence plots, we employ an autoencoder which is used to reduce the dimensionality of these plots while preserving essential features. The latent space embeddings obtained from the autoencoder provide a compact and comprehensive representation of the Recurrence plots, effectively capturing the essential dynamics of the ECG signals for different CVDs. Subsequently, RQA features are extracted from these embeddings. RQA features quantify various aspects of the Recurrence plots, such as the frequency and length of recurrence patterns, and their distribution. These features serve as inputs for ensemble classifiers, which combine multiple learning algorithms to improve classification performance. The integration of these methodologies results in a robust framework for CVD classification, capable of accurately distinguishing between different cardiac conditions. This approach not only enhances diagnostic precision but also leverages the intricate patterns revealed by Recurrence plots to provide a deeper understanding of cardiac dynamics.

### High-precision ECG classification through latent space embeddings of Recurrence plot

**Fig. 2:**
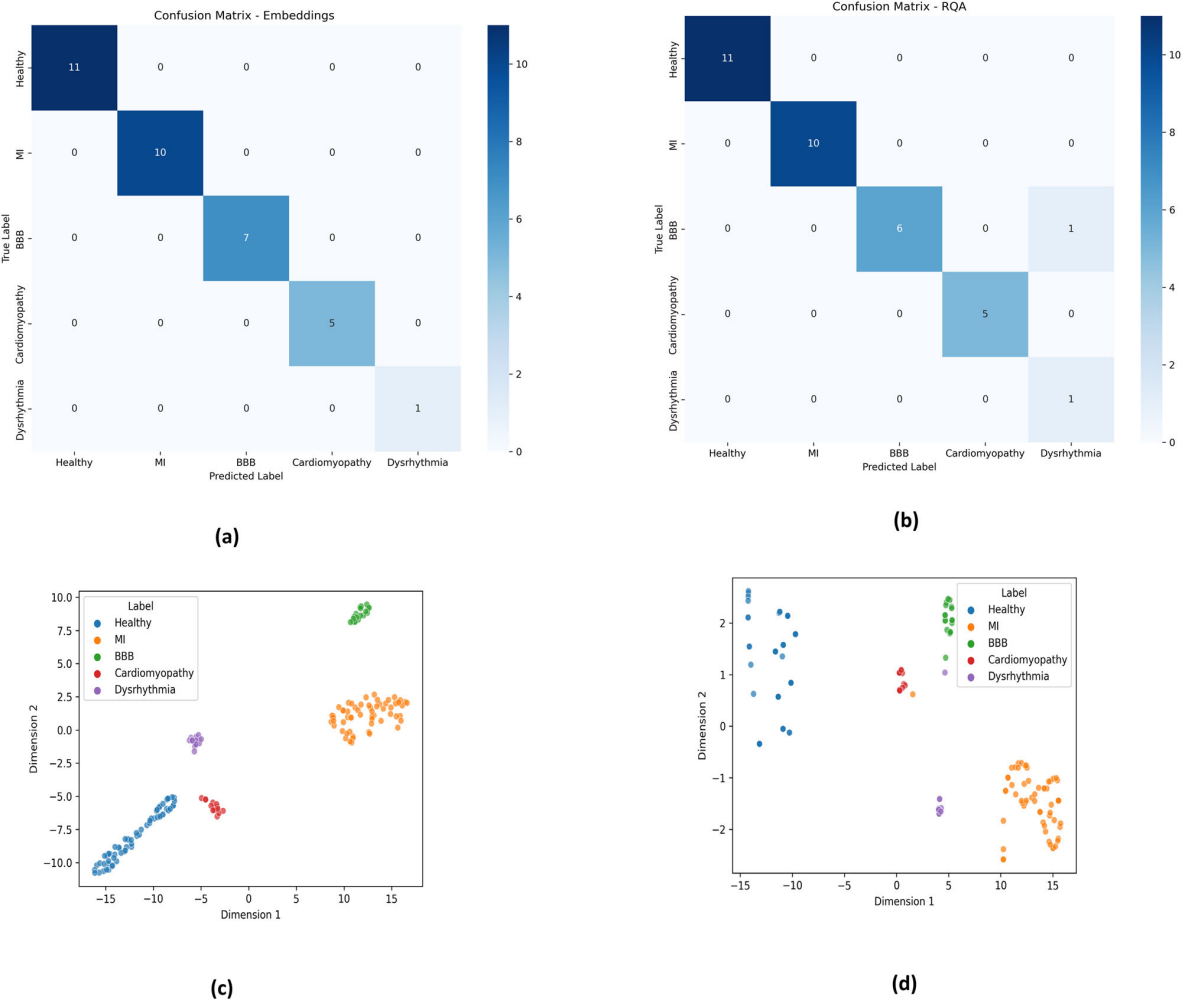
The heat maps of classification performance using. **a** Latent space embeddings of the Recurrence plot and **b** latent space RQA features, are presented in Fig. 2a, b, respectively. The corresponding t-SNE visualizations on the test data are shown in Fig. 2c, d. These figures demonstrate the inter-class separability among different CVDs and HC achieved by the proposed method.

The first approach in this study focuses on utilizing latent space embeddings derived from 2D Recurrence plots of ECG signals. Each ECG channel is transformed into a 224 × 224 Recurrence plot, resulting in a high-dimensional input of 15 × 224 × 224 for each recording. These high-dimensional representations are processed through an autoencoder to reduce dimensionality, producing latent space embeddings of size 14 × 14. These embeddings are then used as input to a custom convolutional neural network (CNN)-based classifier, specifically designed to leverage the extracted features from the Recurrence plots. The classifier achieved a peak accuracy of 100%, demonstrating its exceptional ability to distinguish between different cardiac disorders and healthy controls. The heat map of the confusion matrix, presented in Fig. 2a, provides a visual representation of the classification performance, showcasing the model’s effectiveness in correctly identifying all five classes. Additionally, the t-SNE visualization shown in Fig. 2c illustrates well-separated clusters, further highlighting the clear inter-class separability achieved by this method.

### Latent space RQA-driven insights for prediction of CVDs

**Fig. 3:**
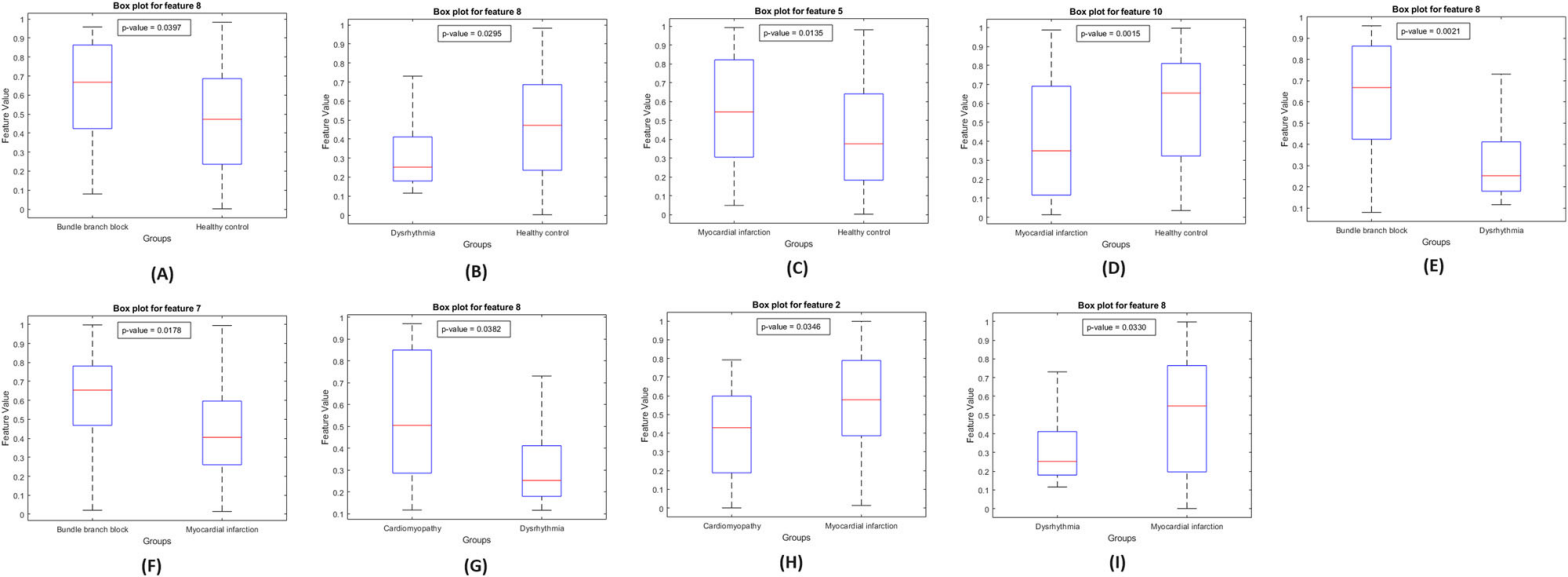
The box plot illustrates the results of the Wilcoxon rank-sum test, conducted with a 95% confidence interval, for pairwise comparisons between the study groups. **A** F8 (Longest vertical line) between BBB and HC, **B** F8 between DR and HC, **C** F5 (Entropy) between MI and HC, **D** F10 (RDR) between MI and HC, **E** F8 between BBB and DR, **F** F7 (TT) between BBB and MI, **G** F8 between CM and DR, **H** F2 (Determinism) between CM and MI, and I F8 between DR and MI.

In the second approach, the study extends the analysis by extracting 10 RQA features from the 14 × 14 latent space embeddings generated by the autoencoder. These features include recurrence rate, determinism, average diagonal line length, longest diagonal line, entropy, laminarity, trapping time, longest vertical line, divergence, and the recurrence–determinism ratio. The details of these features are provided in Section 4.5. To determine which RQA features exhibit significant differences across study groups, the Wilcoxon Rank-Sum test is conducted at a 95% confidence interval. The analysis identified 5 RQA features that exhibited statistically significant differences between different study groups, taken pairwise, with a significance level of *p* < 0.05. The results of this test are depicted in a box plot, as shown in Fig. 3. For classification purposes, the extracted RQA features are used as input to a stacked ensemble classifier. This approach achieved a peak accuracy of 97.05%, further validating the utility of RQA features in distinguishing between various cardiac conditions. The heat map of the confusion matrix, presented in Fig. 2b, visually confirms the classification performance. The corresponding t-SNE plot, shown in Fig. 2d, displays well-separated clusters, reinforcing the effectiveness of using RQA features for classification- as shown in Table 1.

**Table 1:** The five-class classification performance of the proposed methodology is evaluated using two models: (1) the stacked classifier utilizes RQA features, while (2) the CNN utilizes latent space embeddings of Recurrence plot

Models	Classes	Precision	Recall	F1-score	Accuracy
Stacked classifier	HC	1.00	1.00	1.00	
	MI	1.00	1.00	1.00	
	BBB	1.00	0.86	0.92	97.05%
	CM	1.00	1.00	1.00	
	DR	0.50	1.00	0.67	
CNN classifier	HC	1.00	1.00	1.00	
	MI	1.00	1.00	1.00	
	BBB	1.00	1.00	1.00	100%
	CM	1.00	1.00	1.00	
	DR	1.00	1.00	1.00	

The results obtained from this analysis highlight the significant effectiveness of recurrence plot-based methods in accurately classifying various CVDs. The comparison of the proposed method with state-of-the-art techniques is tabulated in Table 2. It is seen that the proposed method outperforms the state-of-the-art methods that utilized the same dataset for CVDs classification. By leveraging the detailed patterns and dynamics captured in recurrence plots, this approach offers a deep and nuanced understanding of the temporal dependencies and structural characteristics inherent in ECG signals. The non-linear, recurrence plot-based analysis establishes a powerful and innovative framework for both clinical practice and research. Its ability to capture and analyze complex cardiac dynamics offers a significant advancement in the field of cardiovascular diagnostics, promising improved outcomes and insights into heart disease.

**Table 2:** Comparison of the proposed study with state-of-the-art methods that used the same PTB dataset from PhysioNet

REF	Year	Subject	Methods	Classifier	Accuracy
^5^	2021	HC, MI,	Data	CNN	76.5%
		CD, STTC,	filtering	with entropy	
		and HYP		features	
^7^	2022	HC, MI,	QRS	Few	93.2%
		CD, STTC,	Extracting	Short	
		and HYP		Learning	
^37^	2021	AR and	Raw	CNN	93.14%
		HC	Input	NCBAM	
^38^	2017	MI and	Beat	ML-CNN	96%
		HC	segmentation		
^39^	2022	MI and	Normalization,	Random	74%
		HC	segmentation	forest	
**RP Based Method**	2024	**HC, BBB, MI,**	**Recurrence Plot**	**CNN Classifier**	**100%**
		**CM, and DR**	**RQA Mesures**	**Stacked Classifier**	**97.05%**

## Discussion

Recurrence plots are traditionally used to infer structural characteristics, detect chaos, and evaluate predictability or unpredictability within time series data. In the context of studying various CVDs, recurrence plots reveal the complex patterns of recurrence within ECG signals, capturing subtle and intricate dynamical behaviors specific to each condition. They are particularly useful as they visualize and quantify the non-linear and non-stationary aspects of ECG signals, aspects often overlooked by traditional linear analysis methods. By uncovering hidden structures, chaos, and predictability within the ECG data, recurrence plots offer a deeper understanding of cardiac dynamics, enhancing the ability to differentiate between different CVDs. While traditional methods focus on analyzing recurrence plots through individual RQA, our approach introduces novelty by combining feature embeddings of these plots with RQA measures derived from the embeddings. This study seeks to develop a more effective technique for classifying different CVDs using ECG data by integrating Recurrence plots and RQA measures derived from autoencoder latent space embeddings of ECG signals. The hypothesis of the study is that the dynamics of ECG signals differ in the presence of cardiac abnormalities. We showed that transforming 1D ECG signals into 2D Recurrence plots, followed by extracting RQA measures from autoencoder latent space embeddings, provides a more informative and discriminative feature set for accurate classification as compared to traditional methods. Furthermore, the proposed study demonstrates superior performance across different types of classifiers, including CNN-based classifiers and stacked ensemble classifiers, thereby validating the robustness and generalizability of the approach.

**Fig. 4:**
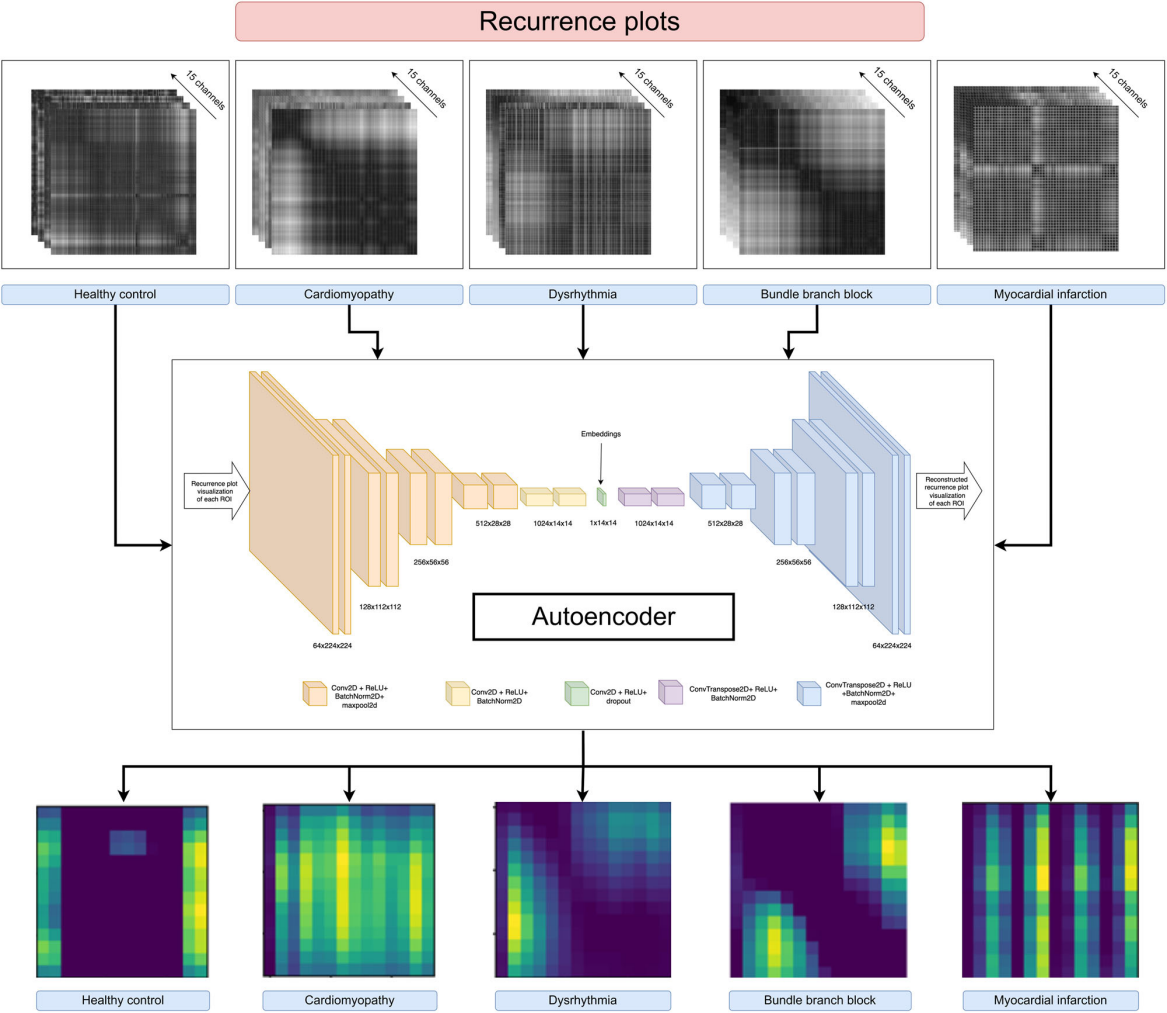
Visualization of autoencoder 2D latent space embedding from Recurrence plots of ECG signal across 15 channels. As seen from this figure, the latent space of the autoencoder showed a clear and distinctive frequency pattern among different CVDs and HC, thereby enhancing interpretability and offering valuable insights into disease-specific characteristics.

The latent space of the autoencoder in Fig. 4 reveals distinct frequency patterns that could be instrumental in interpreting the dynamics of CVDs. Analyzing these patterns in frequency space offers valuable insights into how these conditions evolve and change over time. Future studies could leverage this frequency information of latent space for better interpretability, to enhance diagnostic accuracy, monitor disease progression, and to develop predictive models. The t-SNE plots visually demonstrate the effectiveness of our method. Figure 2c showcases the separability of the feature space spanned by the latent space embeddings, while Fig. 2d displays the separability of the feature space defined by the RQA measures of these embeddings. This visual representation underscores the efficacy of our approach in distinguishing between different cardiac conditions. The recent state-of-the-art classification methods, as detailed in Table 2, have utilized different sophisticated techniques on the same dataset. These methods include the use of CNNs with focal loss, entropy features, and few-shot learning frameworks. In contrast, our method significantly outperforms these existing models. By employing latent space embeddings derived from Recurrence plots, we achieve a perfect classification accuracy of 100%. Additionally, when using the RQA measures of these embeddings, our method attains a high accuracy of 97.05%. This substantial improvement in performance is noteworthy. Specifically, our method surpasses the current models by a margin of 4% when using autoencoder latent space embeddings and by 1% when utilizing RQA measures of these embeddings. These results highlight the robustness and effectiveness of our approach in classifying various CVDs.

The proposed study contributes a novel approach that leverages non-linear analysis of ECG signals through Recurrence plots, autoencoder latent space embedding, and RQA measures to detect various cardiac disorders. The proposed method achieves superior performance compared to existing state-of-the-art techniques. We hypothesize that the dynamics of ECG signals change in the presence of cardiac abnormalities. Low-dimensional feature embeddings combine information across multi-channel ECG data, enabling efficient classification, with differences in latent space patterns among CVDs demonstrating the approach’s effectiveness and promising accuracy. The applicability of the proposed approach lies in its ability to enable early detection of cardiac disorders by capturing subtle ECG dynamics that traditional methods may overlook. The approach supports personalized healthcare by distinguishing between different cardiac disorders, potentially assisting clinicians in tailored treatment strategies. However, the small sample size is a limitation of the present study, which may hinder the generalizability of the results. A larger and more diverse dataset would improve the robustness of the findings. Additionally, the presence of noise in the ECG data could potentially affect the effectiveness of the proposed method. Future work could explore integrating additional non-linear analysis techniques and advanced deep learning architectures, such as transformer models or graph neural networks, to enhance feature extraction and classification accuracy. Expanding the dataset to include a wider range of cardiac conditions and diverse patient demographics, along with implementing real-time processing capabilities, would help validate the generalizability of the proposed approach and practical applicability in clinical settings.

## Methods

The present study aims to classify various cardiac disorders, including bundle branch block, cardiomyopathy, dysrhythmia, and myocardial infarction, as well as healthy subjects, by utilizing latent space embeddings of Recurrence plots and RQA measures. The proposed technique is comprehensively detailed in the following subsections.

### Dataset description

The Physikalisch-Technische Bundesanstalt (PTB) dataset from the PhysioNet is employed in this study^16^. This dataset comprises 549 recordings of standard 12-lead ECG signals from 290 subjects aged between 17 and 85 years. Each record includes 15 channels simultaneously measured signals: the conventional 12 lead (i, ii, iii, avr, avl, avf, v1, v2, v3, v4, v5, v6) together with the 3 Frank lead ECGs(*v*_*x*_, *v*_*y*_, *v*_*z*_). Each signal is digitized at 1000Hz, with 16-bit resolution over a range of ±16.384 mV^16,17^. The diagnostic classes in the dataset include myocardial infarction, cardiomyopathy, bundle branch block, dysrhythmia, myocardial hypertrophy, valvular heart disease, myocarditis, healthy control, and a few miscellaneous subjects. However, due to the limited number of samples, this study excludes data on myocardial hypertrophy, valvular heart disease, and myocarditis. Thus, in the current study, we utilize ECG recordings of myocardial infarction (MI: *n* = 60), bundle branch block (BBB: *n* = 19), cardiomyopathy (CM: *n* = 15), Dysrhythmia (DR: *n* = 14), and healthy control (HC: *n* = 62), obtained from the publicly available PhysioNet database. These four classes (MI, BBB, CM, and DR) are medically interrelated and represent a broad spectrum of cardiovascular conditions that share common pathological mechanisms and clinical implications. For example, patients who experience an MI are at a higher risk of developing both DR and CM^18,19^. Similarly, BBB can be a marker of underlying heart disease, particularly when associated with MI or CM^20,21^. By considering these conditions together, the study captures a realistic and medically relevant spectrum of cardiac abnormalities, making the classification task more representative of the complex, interrelated nature of cardiovascular diseases. Additionally, classifying these conditions alongside HC allows for a better understanding of the diagnostic potential of ECG signal analysis in identifying these common but often overlapping CVDs.

Several studies have demonstrated that downsampling ECG signals to 500 or 250 Hz yields excellent concordance^22–24^. A sampling frequency of 250 Hz is considered acceptable for heart rate variability (HRV) analysis^25^. Therefore, in this study, the original ECG signals are downsampled to 250 Hz. This is to be noted that no other filtering is performed for denoising in order to retain the original characteristics of the signal. The study utilizes the down-sampled raw ECG data for analysis and classification purposes. The entire experiment is conducted on 24 GB A5000 GPU with Keras framework.

### Spatial encoding of ECG using Recurrence plot

To explore the complexity within each ECG signal, our approach employs nonlinear complexity analysis through Recurrence plots^26^. This method is highly effective and widely recognized for visualizing and understanding nonlinear time series. The concept of recurrences was first introduced by Henri Poincaré in 1890, with the Poincaré recurrence theorem stating that certain dynamical systems will eventually return to a state that is arbitrarily close to or exactly the same as their initial state after a sufficiently long but finite period. This fundamental property of many dynamic systems is also observed in various natural processes^27^. In 1987, Eckmann et al.^28^ advanced the visualization of dynamical systems recurrences through Recurrence plots, which were later combined with RQA. RQA is a nonlinear data analysis technique that quantifies the number and duration of recurrences in a dynamical system based on its state space trajectory^29^. This method has been applied to time series from nonlinear deterministic systems such as financial markets and weather forecasting. For example, Fabretti and Ausloos^30^ used Recurrence plots and RQA to identify critical regimes in financial indices, while Peter Martey Addo et al.^31^ employed these tools to uncover hidden patterns and characterize financial cycles during crises. In neuroscience, Bielski et al.^32^ utilized RQA to parcellate the human amygdala, and Kang et al.^33^ applied RQA to analyze the duration, predictability, and complexity of the default mode network time series in schizophrenia studies. In this study, we adopt autoencoder latent space embeddings of Recurrence plots and RQA measures to analyze ECG signals for classifying the considered CVDs. By investigating the distinct patterns revealed in Recurrence plots, we can effectively distinguish between different CVDs, offering valuable insights into the unique dynamics of heart activity for each condition.

### Construction of Recurrence plots

For an ECG signal {*x*_1_, *x*_2_, …, *x*_*N*_} consisting of *N* timestamps, we create *K* state vectors. Each state vector ${\overrightarrow{s}}_{i}$ is an *M*-dimensional vector defined by:

1 $${\overrightarrow{s}}_{i}=({v}_{i},{v}_{i+\tau },{v}_{i+2\tau },\ldots ,{v}_{i+(N-1)\tau })$$

To apply non-linear analysis, the optimal values of the required parameters, embedding dimension (*M*), time lag (*τ*), and number of states (*K*), are determined using the traditional Cao’s algorithm^26^. The Eq. (2) is used to construct the data matrix, represented as ${\mathcal{D}}$ which consists of *K* states stacked together. The dimension of ${\mathcal{D}}$ is *K* × *M*. This matrix ${\mathcal{D}}$ is utilized to define the Recurrence Matrix (${\mathcal{R}}$)^34^.

2 $${\mathcal{D}}={\left[\begin{array}{c}{\overrightarrow{s}}_{1}\\ {\overrightarrow{s}}_{2}\\ \vdots \\ {\overrightarrow{s}}_{n}\end{array}\right]}_{(K\times M)}$$

In ${\mathcal{R}}$, the distance between two state vectors is computed using their Euclidean distance, quantifying their dissimilarity. Hence, for a given tuple (*i*, *j*), the distance between states ${\overrightarrow{s}}_{i}$ and ${\overrightarrow{s}}_{j}$ is the (*i*, *j*)th element of the ${\mathcal{R}}$, given by Eq. (3)^34^.

3 $${\mathcal{R}}(i,j)=\,{\mathrm{dist}}\,({\overrightarrow{s}}_{i},{\overrightarrow{s}}_{j})$$

i.e.,

4 $${\mathcal{R}}={\left[\begin{array}{ccc}\mathrm{dist}({\overrightarrow{s}}_{1},{\overrightarrow{s}}_{1})&\cdots &{\mathrm{dist}}({\overrightarrow{s}}_{1},{\overrightarrow{s}}_{K})\\ {\mathrm{dist}}({\overrightarrow{s}}_{2},{\overrightarrow{s}}_{1})&\cdots &{\mathrm{dist}}({\overrightarrow{s}}_{2},{\overrightarrow{s}}_{K})\\ \vdots &\ddots &\vdots \\ {\mathrm{dist}}({\overrightarrow{s}}_{K},{\overrightarrow{s}}_{1})&\cdots &{\mathrm{dist}}({\overrightarrow{s}}_{K},{\overrightarrow{s}}_{K})\end{array}\right]}_{(K\times K)}$$

Eckmann et al.^28^ introduced a method to visualize the recurrence of states $[{\overrightarrow{s}}_{i}]$ within a phase space. This approach involves projecting high-dimensional phase spaces into two- or three-dimensional spaces for easier visualization. The recurrence of a state at time *x* at a different time *y* is depicted in a two-dimensional square matrix, with both axes representing time^35^. This graphical visualization is known as a Recurrence plot (RP) and is mathematically represented as:

$${{\mathcal{R}}}_{x,y}={{\Theta }}({\epsilon }_{x}-\parallel {\overrightarrow{s}}_{x}-{\overrightarrow{s}}_{y}\parallel ),\quad {\overrightarrow{s}}_{x}\in {{\mathbb{R}}}^{n},\quad x,y=1,\ldots ,N,$$

where Θ is the Heaviside step function, *ϵ*_*i*_ is a threshold distance, and $\parallel {\overrightarrow{s}}_{x}-{\overrightarrow{s}}_{y}\parallel$ is the distance between states ${\overrightarrow{s}}_{x}$ and ${\overrightarrow{s}}_{y}$.

In this study, the ${\mathcal{R}}$ matrix of dimensions *K* × *K* is resized to a shape of 224 × 224 to ensure consistency across all ECG channel signals as well as across all subjects. The Recurrence plot is shown in Fig. 4. The information for a specific channel is represented as a grayscale image of size 1 × 224 × 224.

### Autoencoder architecture

Each ECG channel is represented as a 2D grayscale image of size 1 × 224 × 224. For a subject with *N* channels, the Recurrence plots are stacked while preserving the channel ordering across all subjects. This results in a high-dimensional data point of size *N* × 224 × 224. In our study, with 15 ECG channels, each subject is represented as a 15 × 224 × 224 data point. This single high-dimensional representation thus efficiently fuses information across all the ECG channels. In order to reduce this dimensionality and capture the essential characteristics, we have used an autoencoder to obtain a feature embedding of size 14 × 14. The study utilizes a CNN-based autoencoder to obtain the latent space embedding of the Recurrence plots. The autoencoder processes the input Recurrence plots with a size of 15 × 224 × 224. The convolutional layers of the CNN are designed to capture the essential patterns and features present in the Recurrence plots. The training of the autoencoder involves optimizing a loss function composed of two terms, as shown in Eq. (5). The first term focuses on reconstruction fidelity, ensuring that the reconstructed output is as close as possible to the original input. The second term introduces a penalty based on the mean structural similarity index (MSSIM)^36^, which helps preserve the structural information within the Recurrence plots. To train the autoencoder, we used the Adam optimizer, known for its efficiency in handling large datasets and complex models. The training is performed on 2 × *T*4 GPUs, which significantly accelerated the process. It took approximately 3.5 h to train the autoencoder for 1000 epochs. A batch size of 16 is chosen to ensure stable and efficient training and a train-test split ratio of 0.2 is used to validate the model’s performance and avoid overfitting. The resulting latent space embedding, obtained through this training process, effectively reduces the dimensionality of the original 15 × 224 × 224 Recurrence plots to a size of 14 × 14, while preserving the critical features necessary for subsequent analysis and classification of the ECG signals.

5 $$L(x)=| x-\tilde{x}| +(1-\,{\mathrm{MSSIM}}\,(x,{\tilde{x}}))$$

### Latent space RQA-based feature extraction

RQA is used to quantify the number and duration of recurrences in a dynamical system, based on the trajectory of its state space. In this study, we employ 14 × 14 latent space embeddings obtained from the autoencoder, to extract RQA features. The RQA values derived from these latent space embeddings are then used for classification purposes. From the latent space embeddings, the current study utilizes a total of 10 RQA features for classification. These features are described in detail below:

F1. *Recurrence rate (RR)*: The percentage of recurrence points in a Recurrence plot (RP) that corresponds to the correlation sum. RR states the recurrence probability of a specific state. A higher value in RR suggests more frequent state recurrences, often associated with more regular or deterministic systems.

$${{RR}}=\frac{1}{{N}^{2}}\mathop{\sum }\limits_{x,y=1}^{N}{R}_{x,y}$$

F2. *Determinism (D)*: The percentage of recurrence points forming diagonal lines. *P*(*len*) is the histogram of the lengths *len* of the diagonal lines. *D* provides predictability for the dynamic system. A deterministic process has a Recurrence plot with relatively few single dots and numerous lengthy diagonal lines, whereas white noise has only single dots and very few diagonal lines. A high value in *D* implies the system’s behavior is deterministic, showing predictability and structured patterns over time.

$$D=\frac{\mathop{\sum }\nolimits_{len = le{n}_{\min }}^{N}lenP(len)}{\mathop{\sum }\nolimits_{len = 1}^{N}lenP(len)}$$

F3. *Average diagonal line length* $({\bar{d}}_{L})$: The average length of the diagonal lines. It provides the predictability time of the dynamical system. Longer diagonal lines suggest longer predictable periods, indicating that the system exhibits more extended predictable dynamics.

$$\bar{{d}_{L}}=\frac{\mathop{\sum }\nolimits_{len = le{n}_{\min }}^{N}lenP(len)}{\mathop{\sum }\nolimits_{len = le{n}_{\min }}^{N}P(len)}$$

F4. *Longest diagonal line* (${d}_{{L}_{\max }}$): The length of the longest diagonal line. Longer (${d}_{{L}_{\max }}$) values indicate more persistent deterministic behavior.

$${d}_{{L}_{\max }}=\max \{le{n}_{x}\,| \,x=1,\ldots ,{N}_{len}\}$$

F5. *Entropy (H)*: The Shannon entropy of the probability distribution of the diagonal line lengths *p*(*len*). Entropy represents the complexity of the deterministic structure in the system. Higher entropy indicates more complexity and less predictability, reflecting a mix of different pattern lengths.

$$H=-\mathop{\sum }\limits_{len=le{n}_{\min }}^{N}p(len)\ln p(len)$$

F6. *Laminarity (LAM)*: The percentage of recurrence points forming vertical lines.*p*(*vl*) is the histogram of the lengths *vl* of the vertical lines. Laminarity gives the value for the amount of laminar phases in the system (intermittency). A high value in *LAM* suggests that the system spends more time in specific states, indicative of intermittent or laminar phases.

$${{LAM}}=\frac{\mathop{\sum }\nolimits_{vl = v{l}_{\min }}^{N}vlP(vl)}{\mathop{\sum }\nolimits_{vl = 1}^{N}vlP(vl)}$$

F7. *Trapping time (TT)*: The average length of the vertical lines. It is related to the laminarity time of the dynamical system. Longer trapping times suggest more persistent state trapping.

$$TT=\frac{\mathop{\sum }\nolimits_{vl = v{l}_{\min }}^{N}vlP(vl)}{\mathop{\sum }\nolimits_{vl = v{l}_{\min }}^{N}P(vl)}$$

F8. *Longest vertical line* (${V}_{\max }$): The length of the longest vertical line.

$${V}_{\max }=\max \{v{l}_{x}\,| \,x=1,\ldots ,{N}_{vl}\}$$

F9. *Divergence (DIV)*: The inverse of ${L}_{\max }$, related to the KS entropy of the system, i.e., the sum of the positive Lyapunov exponents. The reciprocal of the maximal length of the diagonal lines is an estimator for the positive maximal Lyapunov exponent of the dynamical system as the length of the diagonal lines is related to the time how long segments of the phase space trajectory run parallel or on the divergence behavior of the trajectories. Longer trapping times suggest more persistent state trapping.

$${{DIV}}=\frac{1}{{d}_{{L}_{\max }}}$$

F10. *Recurrence-determinism ratio (RDR)*: It is the ratio between *D* and *RR*. A higher value in ratio suggests a more deterministic system relative to its recurrence density.

$${{RDR}}={N}^{2}\frac{\mathop{\sum }\nolimits_{len = le{n}_{\min }}^{N}lenP(len)}{{\left(\mathop{\sum }\nolimits_{len = 1}^{N}lenP(len)\right)}^{2}}$$

### Classification

To classify various CVDs, we employ two distinct classifiers: a custom CNN-based classifier and a stacked classifier. Each classifier leverages the latent space feature embeddings derived from the Recurrence plots of ECG signals.

1. *CNN-based classifier*: The first classifier is a custom CNN-based model designed specifically for this task. The input to this CNN classifier is the set of latent space feature embeddings obtained from the Recurrence plots. The architecture of this network includes three convolutional layers, which are responsible for capturing the essential patterns and features within the latent space embeddings, and six fully connected layers, which are used for prediction, refining the extracted features, and making the final classification. The network is trained using a cross-entropy loss function, which is well-suited for classification tasks. The Adam optimizer is employed to enhance the training process by efficiently updating the network’s weights.
2. *Stacked classifier*: The second classifier is a stacked classifier, an ensemble method that combines the strengths of multiple classifiers. The stacked classifier operates as follows:
  (i) Base models: The first layer of the stacking ensemble consists of three machine learning-based classifiers: support vector machines (SVM), XGBoost, and RUSBoost.
  (ii) Meta-classifier: The second layer, also known as the meta-model, is a logistic regression model. The role of the meta-classifier is to combine the predictions made by the base models to produce the final classification. Each base model is independently trained on the training dataset. This diversity allows each model to capture different patterns and relationships within the data that might be missed by a single model. After the base models have been trained, their predictions on the training dataset are used as inputs for the meta-classifier. The meta-classifier learns how to best combine these predictions to make the final decision. The input to the stacked classifier is the RQA-based features of the autoencoder latent space embeddings derived from the Recurrence plots. The RQA values capture the intricate dynamics and structural information of the ECG signals, providing a rich feature set for the classifiers. By utilizing both a custom CNN-based classifier and a sophisticated stacked classifier, we leverage the strengths of deep learning and ensemble methods to achieve robust and accurate classification of cardiac disorders. The custom CNN excels in capturing spatial features from the Recurrence plots, while the stacked classifier benefits from the diverse perspectives of multiple machine learning models, combined into a powerful meta-classifier. This dual approach ensures a comprehensive analysis and enhances the reliability of our classification system.

## Data Availability

Sequence data that support the findings of this study have been deposited in the PTB Diagnostic ECG Database with the primary accession code/link https://physionet.org/content/ptbdb/1.0.0/.

## Code availability

The code of this study is available on GitHub.
